# (Re)defining legitimacy in Canadian drug assessment policy? Comparing ideas over time

**DOI:** 10.1017/S1744133121000013

**Published:** 2021-10

**Authors:** Katherine Boothe

**Affiliations:** McMaster University Ringgold Standard Institution – Political Science, 1280 Main St West Hamilton, Hamilton, OntarioL8S 4L8, Canada

**Keywords:** Comparative qualitative analysis, evidence-based policy, health technology assessment, legitimacy, policy paradigms, public and patient involvement

## Abstract

How do experts judge the legitimacy of technical policy processes, and do their ideas change as these processes are opened to other stakeholders and the public? This research examines the adoption of public and patient involvement in pharmaceutical assessment in Canada. It finds tensions between scientific legitimacy that prioritizes rigor and objectivity, and democratic legitimacy that values inclusion and a broader range of evidence. In response to policy change, experts incorporate new ideas about democratic inputs and processes, while maintaining scientific policy goals. The research responds to calls for more precise measurement of ideas and ideational change and more evaluation of public and patient involvement in health policy. It helps us understand the significance of, and limits to, ideational change among experts in health policy domains that are highly technical and publicly salient. Understanding the way democratic and scientific legitimacy are negotiated in policy decisions has a wide applicability in health, but is particularly relevant during a global pandemic when evidence is being generated rapidly, decisions must be made quickly, and these decisions have a significant, immediate effect on the lives of all citizens.

## 1. Introduction

In Canada, pharmaceuticals are an anomaly in the public health system. Governments provide universal, comprehensive public hospital and medical insurance on a relatively uniform basis nation-wide, but unlike all other countries with a broad public health system, outpatient pharmaceuticals are not included. Instead, various limited public pharmaceutical programs are offered by federal, provincial and territorial governments. These programs ration resources both by restricting beneficiary eligibility (often by age or income), and by limiting the therapies eligible for reimbursement by creating a formulary, or list of covered drugs (Brandt *et al*., 2018): this paper focuses on the later.

Public drug plans in Canada make formulary decisions using health technology assessment (HTA) methods. HTA uses a range of clinical, economic, legal and ethical evidence to determine which new drugs should be publicly funded, based on their clinical and cost effectiveness. The methods are implemented by independent drug advisory committees made up of health care professionals, clinical epidemiologists, health economists and other technical experts, which make non-binding recommendations to public drug plans.

This paper examines the Ontario drug assessment process and both pan-Canadian drug assessment processes (the Common Drug Review and the pan-Canadian Oncology Drug Review). Since 2006, each of these drug advisory committees has included patients or members of the public. I describe these individuals as ‘lay members’ of the committee to distinguish them from members with specific technical or clinical expertise. All these committees accept input from patient groups when new drugs relevant to the group's condition are considered for reimbursement. The lay members' role on all committees is to summarize and present patient submissions at committee meetings, to participate in deliberation with technical members of the committee, and to vote on recommendations. Public and patient involvement was introduced to address perceived legitimacy deficits in Canadian drug assessment processes and in response to pressure from patient advocacy groups (Standing Committee on Health, 2007a; MacPhail and Shea, 2017: 50). However, the perceived deficit was not in the eyes of existing technical members, who report being satisfied with the process around the time public members were added to the committee (Ekos Research Associates Inc., 2005; Standing Committee on Health, 2007a). Instead, the legitimacy challenge came from outside the process.

This paper takes the adoption of public and patient involvement in some Canadian drug advisory committees starting in 2006 as an opportunity to study changing ideas about legitimacy. It asks how policy actors respond when faced with an institutional change that challenges their ideas about their work and professional identity. In particular, it asks whether and how committee members' understandings of legitimacy have changed since the introduction of public and patient involvement by considering the potentially conflicting paradigms of scientific and democratic legitimacy.

The research finds that there has been some change in how technical members of drug advisory committees express ideas about legitimacy in drug assessment. Over time, technical members as a group have maintained an orthodox scientific understanding of the goals of drug assessment, while referencing newer ideas about democratic processes and inputs. Thus, the change observed in technical members' ideas over time is best characterized as the creation of a new, hybrid paradigm that contains elements of both scientific and democratic legitimacy, and it still weighted to scientific standards, especially when it comes to goals for drug assessment.

This research contributes to critical assessments of evidence-based policy making (Daston, 1995; Lehoux *et al*., 2008; Ducey *et al*., 2015). It suggests that ‘evidence’, uncritically defined, is not a silver bullet to better policy outcomes, or a solution to legitimacy conflicts when it comes to the distribution of scarce resources. It responds to Ducey *et al*.'s call for research into ‘when and why different types of evidence are accepted in principle and practice; [and] whether and how values might alter the definitions of evidence’ (Ducey *et al*., 2015: 23). The possibility of tensions between the beliefs and practices of technical drug assessment and public and patient involvement has been raised in the literature (Gauvin *et al*., 2010; Menon and Stafinski, 2011: 78; Berglas *et al*., 2016) but until now has not been studied across multiple methods of public and patient involvement or multiple drug advisory committees. It has immediate practical significance in Canada, where discussions of expanded public drug insurance are increasingly prominent, and where the pharmaceutical industry is increasingly vocal about what it sees as the problems of limited formularies (Innovative Medicines Canada, 2018). It is relevant to other countries that mandate public and patient involvement in drug assessment (Staley and Doherty, 2016; Jones and Pietilä, 2017). Finally, a better understanding of the tensions between scientific evidence and public and patient involvement is critical in the current global pandemic as governments and agencies work to synthesize rapidly changing evidence and produce effective and acceptable public health policies, whereas patient advocates and members of the public demand a meaningful role in the discussion (Murphy *et al*., 2020; Richards and Scowcroft, 2020).

This paper also contributes to literature on paradigm change, and the links between the institutional and ideational change (Hall, 1993; Mahoney and Thelen, 2010; Carstensen, 2016). Ideas have been found to drive political behavior (Jacobs, 2009), but there are important gaps in our understanding of how ideas change (Berman, 2001; Carstensen and Schmidt, 2015). This research asks whether actors' ideas change as institutions change around them, and how that change can be measured and characterized. Understanding the nature of ideational change helps us understand both the limits of change and its significance. A key finding of this research is that gradual, non-radical change can occur in the absence of wholesale paradigm change, and that it has a meaningful impact on how actors relate to a policy process.

## 2. Data and methods

This paper analyzes the ideas of technical members during the early implementation of public and patient involvement (2005–2012), and compares them to technical members' ideas during a later period when public and patient involvement was well-established (2016–2019). To do so, it draws on assessments of the Common Drug Review published between 2005 and 2012 (Ekos Research Associates Inc., 2005; Standing Committee on Health, 2007b; SECOR 2011; Lague and SECOR 2012; SECOR 2012); interviews with 13 current or former drug advisory committee members and one patient group representative conducted between 2014 and 2019, and post-interview participant feedback. The reports represent all publicly available evaluations of the Common Drug Review since its inception: there are currently no similar, publicly available evaluations published since 2012 that could complement the interview data used for the second time period. Other Canadian drug advisory committees did not undergo the same process of scrutiny and self-reflection in the early days of public and patient involvement, but the reports include some comparisons to other Canadian committees (Lague and SECOR, 2012). The reports are a valuable source of evidence about participants' ideas because they are explicitly focused on evaluating the drug assessment process and the role of public and patient involvement in it, and include both summary statements and direct quotations from technical committee members. A number of questions posed in the reports are repeated during the later interviews.

One pilot interview was conducted in 2014; the rest were conducted in 2016, 2018 and 2019 as part of a larger set of interviews with the full range of participants in drug assessment (lay members, officials and patient advocates). The research also included a member-checking process. Interviewees were sent a brief synthesis of the initial analysis with a request for their comments. This strategy allows for more nuanced analysis: even if participants disagree with some findings, the nature of their disagreement can improve the researcher account (Turner and Coen, 2008; Birt *et al*., 2016). The participant feedback form was sent to all 13 technical participants. Twelve responses were received, eight with substantive comments.

This paper focuses on technical committee members because they are responsible for implementing public and patient involvement in HTA and negotiating how it occurs, and therefore have a significant role in the success of public and patient involvement. According to May *et al*. (2003: 708), their role is one of ‘uncommitted outsiders … charged with the production and processing of legitimating knowledge about “effectiveness” and “utility”’, in this case, of new drugs. This may make it challenging for technical members to accept alternate ideas about legitimacy, and research has found they may be particularly likely to hold ideas about HTA, evidence and objectivity that are challenged by public and patient involvement (Greenhalgh and Russell, 2006; Ducey *et al*., 2015).

The research does not attempt to trace change in individuals' ideas during their tenure on drug advisory committees. Instead, it considers evidence about the views of technical members as a group over time. It asks whether there is evidence of different ideas among members in recent years, although it cannot determine whether new ideas are a consequence of individuals changing their minds, or new members joining the committee.

The reports and interview transcripts were coded using Nvivo 11 software. The coding scheme was developed using an open coding method to identify concepts and common themes. As the transcripts were read and reread, topics key to participants' understanding of HTA and public and patient involvement began to emerge, and analytic memos were developed to help clarify patterns. Memos were developed on themes related to goals for drug assessment, goals for public and patient involvement and different terms related to legitimacy. The memos also explored roles for patient and public members, and questions about the quality of evidence used in the drug assessment process. Substantive participant feedback was also coded and added to the memos. Where it is quoted in the analysis, the interviewee's original code is followed by an ‘f’ to indicate this was feedback gathered in 2019.

## 3. Conceptualizing legitimacy and measuring change

There may be multiple, and at times conflicting, goals for public and patient involvement in HTA (Rowe and Frewer, 2000; Mitton *et al*., 2006; Knaapen and Lehoux, 2016; Rosenberg-Yunger and Bayoumi, 2017). Most debates about goals for public and patient involvement come back to the issue of legitimacy: what makes decisions about the allocation of scarce health resources acceptable (and to whom)? A key motivation in adopting public and patient involvement in Canadian drug assessment was to bolster the *democratic* legitimacy of the process. Drug assessment is highly technical and, in the early 2000s, was increasingly seen as too opaque by stakeholders, particularly patient advocacy groups. However, the initial implementation of public and patient involvement in Canada did not address tensions between what existing participants view as legitimate HTA, and what patients and members of the public might understand as legitimate. This tension illustrates the challenge of studying changes to actors' deeply held beliefs as separate from changes to policy. Béland and Cox (2015) have found that ideas (like ‘democratic legitimacy in drug assessment’) that can be understood in different ways and tend to have a strongly positive association can be particularly important drivers of policy change. This may help us understand why public and patient involvement was adopted, but it cannot explain stability and change in actors' interpretation of the goals of drug assessment or the key markers of legitimacy. This paper characterizes potentially competing views as *scientific* and *democratic* versions of legitimacy, and argues that they constitute paradigms for understanding how drug assessment should work (Hall, 1993).

The concept of a policy paradigm helps break out different types of ideas related to drug assessment to see how they affect actors' interpretation of policy problems and solutions. What are the overarching goals of drug assessment? What types of analytical and deliberative processes are appropriate? What are acceptable inputs, both in terms of information and participants? This paper differs from Hall (1993) by arguing that paradigms are not necessarily complete and coherent, and by arguing that paradigms may evolve rather than be replaced (Oliver and Pemberton 2004; Kern, Kuzemko and Mitchell 2014; Carstensen 2016). If competing paradigms are not mutually exclusive, or actors can hold incoherent ideas about a single policy area, it becomes crucial to identify how different components of a paradigm change at different rates and with different implications for actors' behavior.

Abelson *et al*. (2007) categorize the goals of public and patient involvement as process-related, to ‘improve the legitimacy of decision-making’; or instrumental, to produce ‘better quality decisions’ that ‘reflect [patient groups' and publics'] preferences and values’ (Abelson *et al*., 2007: 40). The public policy literature on legitimacy similarly distinguishes between legitimacy related to inputs (democratic participation), throughputs (fair and transparent processes) and outputs (performance and effectiveness of policy outcomes) (Skogstad, 2003; Montpetit, 2008; Schmidt, 2013).

It may seem obvious to link the technical aspects of drug recommendations to instrumental, outcome-oriented goals to create definition of *scientific legitimacy*, whereas concerns about democratic participation and transparent process define a procedural concern with inputs and throughputs – *democratic legitimacy*. However, this would obscure more complex paradigms of legitimacy where scientific and democratic legitimacy are both concerned with process *and* outcomes, but value these concepts differently.

First, consider the components of a scientific conception of legitimacy. The HTA literature discuss legitimate outcomes or goals in terms of the efficient and equitable allocation of health resources (Abelson *et al*., 2007; Drummond, 2013). Output legitimacy comes from ‘good’ recommendations that ensure efficacious drugs are available to patients who will benefit from them, within the constraints of a publicly-funded system.

Defining scientifically legitimate drug assessment is also linked to inputs and process, but here it is focused on the quality and types of evidence used and way it is analyzed. Ducey *et al*. (2015: 5) interview technical experts involved in HTA and find that, in terms of evidence, ‘aggregate, population-level data was preferred, with studies using control groups and randomisation preferred over cohort studies, and particularistic data such as case studies avoided if possible’. These norms about quality of evidence – that are themselves value-laden (Greenhalgh and Russell, 2006; Ducey *et al*., 2015) – are common to many sciences, and exclude the type of information that might be provided by public or patient input. In terms of data analysis, HTA practitioners have been found to value objectivity, independence, and a standardized and transparent processes (Rosenberg-Yunger and Bayoumi, 2014; Ducey *et al*., 2015). The value technical experts place on these ideas often runs very deep: Ducey *et al*. (2015) find they form a ‘moral economy’ (citing Daston 1995). This is a set of values and beliefs that define how individuals approach different types of evidence and come to define their professional identities. In coding reports and interviews, I used terms such as *quality of evidence*, *objectivity* and *tradeoffs* to capture ideas about scientific legitimacy.

Democratic legitimacy is also concerned with inputs and process: where data come from and how it is analyzed. However, rather than scientific rigor, it prioritizes inclusion – witness the use of the phrase ‘nothing about us, without us’ by patient advocacy groups (Chu *et al*., 2016). In coding, I use terms such as *accountability*, *fairness*, *representation* and *transparency* to capture ideas about democratic legitimacy. Assessing democratic legitimacy is complicated by the fact that it is also concerned with outcomes, and its goals may differ from a scientific paradigm. For example, although patient groups note a need for inclusion and transparent processes, they also care deeply about the outcomes of drug assessment, particularly positive listing recommendations. One patient advocate described how clinical efficacy is evaluated and then noted,

as a patient that lives with the disease, there's no way to predict what molecule I'm going to best respond to… [a drug] might not stack up in a population…but from a patient perspective, if it helps one or two patients that haven't responded to everything else out there…then it's a really difficult thing (PAT13).

This raises questions about the audience for legitimate drug assessments, as experts, patients and the general public may understand and value outputs differently. This paper focuses on legitimacy *in the eyes of drug advisory committee members*, and asks whether committee members focus exclusively on scientific legitimacy, or whether they acknowledge a role for democratic legitimacy in HTA. It assesses the possibility of change in technical members' ideas by determining whether phrases and concepts associated with democratic legitimacy appear in interviews but not the earlier reports, or if they appear more frequently in interviews vs reports.

The research does not anticipate paradigm replacement. Following Hall (1993), it expects that changes to goals will be the most difficult, and that ideas about scientific legitimacy will still shape technical members' work. However, Kern *et al*. (2014) find that processes of ideational change can be based on ‘multiple perspectives’ and that old ideas may persist alongside new. Similarly, Carstensen (2016) argues for a more agency-centered view of ideational change, noting that actors can solve new problems through a pragmatic assemblage of old and new ideas that may not be logically compatible, a process he describes as bricolage. The research looks for evidence of this type of bricolage, or two paradigms of legitimacy functioning together. For technical members, acceptance of democratic conceptions of legitimacy might mean they acknowledge a broader range of goals for drug assessment even as they prioritize scientific goals, and accept different policy processes and inputs.

## 4. Findings

The paper first analyzes evidence of technical members' ideas about HTA and public and patient involvement available in studies conducted soon after the adoption of public and patient involvement. It finds that at this time, technical members acknowledged a need for more public involvement and greater transparency, but expressed serious concerns about the ways public and patient involvement might detract from the scientific legitimacy of the process.

Next, the paper presents evidence from interviews and finds some changes in the way technical members talk about legitimacy over time. As a group, technical members interviewed since 2016 are more likely to acknowledge democratic conceptions of legitimacy and place less emphasis on conflicts between scientific and democratic legitimacy than technical members in the period covered by the reports. However, there is still a tendency for technical members to attempt to fit public and patient involvement into scientific norms (e.g. patient input would be more valuable if it were collected in a more rigorous way), and there are ongoing concerns about objectivity and the potential for bias in patient input.

### 4.1 Technical members' views of democratic and scientific legitimacy, 2005–2012

Before public and patient involvement was implemented, technical members defined the goals of drug assessment in scientific terms, and were satisfied with how these goals were being met. Technical members were reported to think that the existing drug assessment process was consistent, rigorous, fair, objective and transparent (Ekos, 2005).

Both technical members and drug plan representatives were reported to ‘feel the [committee's] recommendations are clear, relevant, evidence-based and unbiased. [Committee] members interviewed noted that the recommendations are good and getting better and more standardized as a result of feedback from drug plans and industry’ (Ekos, 2005: 17).

As public and patient involvement was being implemented, technical members acknowledged some support for changes to inputs and process in line with democratic ideas about legitimacy. They expressed a need for ‘active, meaningful input and participation’ from public; the main barriers identified were ‘manufacturers’ need to protect confidential or proprietary information’ (Ekos, 2005: 11 and 44; Standing Committee on Health, 2007a).

This support for changes to inputs and process was accompanied by a consistent focus on goals defined in terms of scientific legitimacy. In 2007, a CADTH expert reviewer told the parliamentary committee that although existing drug assessment process was ‘very rigorous’, it could be improved by additional information about quality of life, provided by patients (Standing Committee on Health, 2007a, Dr Janis Miyasaki). In 2012, other CADTH reviewers were quoted as valuing patient input as a way to gather better information about health outcomes and potentially produce better recommendations, saying ‘The patient input information simply allows us to be more confident in stating that a given [therapeutic] outcome is of importance to patients, rather than speculating that it is’ (SECOR, 2012: 12).

Some technical members also raised concerns about the risk of bias in patient advocacy groups and the challenges of incorporating new types of information into an evidence-based process. Three technical members interviewed ‘feel there is a need for more public input, but not necessarily from patient advocacy groups’ (Ekos, 2005: 10). A CADTH reviewer noted that the ‘CDR [Common Drug Review] does not, nor is it intended to, assess the ethical or social issues related to drugs approved or listed. I'm not certain that the CDR can incorporate these issues and still remain evidence-based’ (Ekos, 2005: 11), suggesting a perception of conflict between ethical or social considerations and scientific evidence.

Committee members expressed concerns about the objectivity of patient input, saying it risked being ‘another selling avenue for companies’ (Lague and SECOR, 2012: 12), reflecting concerns about the funds many patient groups receive from pharmaceutical manufacturers. They also saw a clear divide between ‘objective clinical cost data’ and patient input. One member was ‘Not sure that patient evidence should be given much weight versus objective clinical cost data. It is inherently subjective and biased: no patient or patient group will ever not want access to a new drug’ (Lague and SECOR 2012: 12).

This suggests that in the early years of public and patient involvement, technical members were mainly focused on how these changes affected the scientific legitimacy of the committees' work. They acknowledged the policy change responded to a perceived democratic legitimacy deficit, and a particular focus on transparency concerns. Critics of the existing process called for the committee to be more transparent, but committee members were reported to think that the main barriers to increased transparency lay with pharmaceutical manufacturers, not HTA processes. There was some cautious consideration of the ways that new inputs and processes might improve the committee's recommendations, but goals continued to be defined in terms of scientific legitimacy. There was also a good deal of concern about the objectivity of this new information, and the potential conflict it presented for a rigorous scientific process.

### 4.2 Technical members' views of democratic and scientific legitimacy, 2016–2019

With the reports as a backdrop, it is possible to analyze interviews with technical members and determine whether there are changes to how these participants, as a group, talk about the process of drug assessment, its inputs and its goals. The research finds the most uptake of new ideas about democratic legitimacy in discussions of drug assessment processes. Ideas about inputs are more contested: I observe new ideas about democratic legitimacy alongside existing ideas about scientific inputs, suggesting this is a key site for bricolage. Technical members' ideas about the goals of drug assessment remained consistent across the reports and interviews and focused on scientific legitimacy.

#### 4.2.1 Legitimate processes: the role of lay members

Ideas about the importance of democratic processes often arose in interviews with reference to lay members of drug advisory committees. One technical member suggested that the role of lay members ‘is actually to legitimize a liberal democratic process… We live in a liberal democracy where we have policy makers who are accountable to people, and they're used to stakeholder input’ (TEC07).

Others described a more substantive democratic role for lay members in terms of ensuring patient values are accounted for and communicating recommendations to the public.

I think that the presence of [lay] members is essential to really make sure that when we go out with a recommendation, it is clear to the public why that recommendation was made. So I think the improvement in the process has been amazing comparing to the early days [before public and patient involvement] because …it was just a black box (TEC17).

If you don't have a [lay] member, even if you address the patient issue in your minutes or whatever letter goes out… there could always be a critic that says: how do I really know you're emphasizing patient values, there's no patient on your committee, there's no public member. And so I agree with that criticism, and I think that having someone there, from an optics point of view, makes a lot of sense (TEC05).

Although the reference to having lay members for ‘optics’ sounds tokenistic, later in the interview, the individual commented that ‘The patients need a voice. And we need to listen to them…whether that influences us one way or the other on a given day, that's really up to the decision-maker, but to not have them at the table makes no sense’ (TEC05). In the participant feedback, another interviewee raised concerns that this comment about ‘optics’ suggested that lay members had a merely symbolic role. The interviewee emphasized that

Having a [lay] member is important from more than one perspective, including the value they bring and the optics of having them as members…hearing about a perspective [from a written submission] is not the same as having someone present and participating actively in the discussions (TEC18f).

Technical members acknowledged a need for democratic participation as an accountability mechanism, and lay members were generally seen as fulfilling this role. Technical members of one committee commented that lay members were ‘fully integrated’ into the committee (TEC10, TEC09).

#### 4.2.2 Legitimate inputs: representativeness

The legitimacy of new inputs to drug assessment was more contentious. Most interviewees were primarily concerned with the content of patient input, but interviewees also reflected on questions of *who* should be involved, raising issues of inclusion that are key in democratic conceptions of legitimacy (Sandman, Bond, and Hofmann, 2017). The interviewer asked whether lay members of the committees are representative, and if so, who they represent. This question was intended to probe conceptions about legitimate inputs, and interviewees' responses suggested that some considered this issue in democratic terms. Technical members did not necessarily see lay members as representative, noting the challenges of having two lay members per committee represent a broad range of interests (TEC18, TEC19, TEC21 and TEC23). Technical members also expressed concerns about whether patient advocacy groups adequately represented the full range of patient interests, or whether patient input was able to communicate social values (TEC07, TEC21, TEC23 and TEC04f). One technical member explained that even though lay members might not be representative, they were still legitimate, noting that others might have had a very different lived experience of [illness], but they [lay members] are legitimate insofar as they have they have walked that journey pretty intimately and emotionally either in the context of their own lives…or someone that they loved (TEC19).

Other technical members commented on the demographic homogeneity of committee members. One noted that the majority of lay members were white and male, and for that reason, ‘they're obviously not going to represent all segments of society’ (TEC21). Another pointed out that although it is unrealistic to expect two lay members on a committee to be entirely representative, the fact that lay members are mostly white, more educated and of higher socioeconomic status means ‘it's worth asking why that is different from all of the patient advocacy organizations and also different from the general population’ (TEC23). These are important critiques in themselves, but they also point to a democratic paradigm for assessing the legitimacy of inputs: it is not only the scientific rigor of inputs that matters, but also who is included.

#### 4.2.3 Legitimate inputs: quality of evidence

There was a clear consensus in interviews on what counts as acceptable technical evidence, and consensus on the role of HTA in ensuring that the best available evidence was used when making drug listing decisions (TEC09, TEC07 and TEC19), and this was consistent with the earlier reports.

The importance of scientific norms around clinical data is sometimes identified as a barrier to accepting patient input as evidence. One technical member described this tension:

I think a lot of people around a committee are very technical, and they want to see good technical clinical epidemiologic information. And when a case account comes of, I took the drug and it was wonderful, I think in people who are quite analytic and jaded, they'll say, that's not really evidence of anything (TEC07).

Another suggested that members' expertise and training affected their view of evidence, saying what [methodologists] consider evidence is a little broader, I would imagine, than what a traditional evidence-based medicine person would consider as evidence…anecdotal evidence is still… It's not ideal, but it tells us a lot. Because you can have a clinical trial that says this drug is wonderful, but then if the patients are saying we're not really concerned about what you've measured in this clinical trial, then it's really important to know that (TEC06).

Other technical members acknowledged the tension between orthodox scientific evidence and patient input, but suggested that the views of the committees on this were starting to change. It is notable that participants themselves reflected on their experience of changing ideas, as this acknowledges that previous views about legitimacy may have gaps.

If you look at patient evidence, which one could consider *not* to be evidence, but which affects the committee, it will be interesting to see how this evolves…RCT [randomized controlled trials] has been king for a long time, but there is a move to consider other types of evidence (TEC18).

Another noted that when the expert committee was first set up, we always had this idea that… this kind of committee would really look only at straight evidence, the assessment of the pharmacoeconomics and the clinical trials, efficacy of the drug… so, back in the day, it [patient input] wasn't really valued because it was always thought that ‘well it's going bring some anecdotal evidence that we cannot really take into consideration’. So, it was almost a given that patients would be excluded. There's been an evolution…it [patient input] brings an important perspective (TEC17).

A number of technical members also reported concerns with public and patient input and these issues were framed in terms of a failure of patient input to conform to scientific norms of legitimacy. The ability to judge the quality of patient input remains a key concern. As one technical member noted,

I don't think we have a handle on…what constitutes really robust patient and public input in the context of drug policy making. Whereas there's much more consensus on – even if it's being challenged – on hierarchies of evidence in the clinical and economic worlds (TEC19).

The idea that the quality of patient input was variable and difficult to assess was expressed by a number of interviewees, and was reinforced in participant feedback where an interviewee commented that ‘there seemed to be an increase in acceptance of the need for patient input but concerns over what that input should be’ (TEC06f).

So if it's the clinical world…that's with reference to evidence-based medicine paradigms, [and] from the world of economic evaluation, it's in line with standard guidelines for conducting economic evaluation. And we talk about uncertainty related to the evidence and those contacts…We don't really talk about quality with respect to the patient input (TEC19).

there are ways of grading the clinical trial data…and for economics, certainly that also exists. I would value having that for the social values, patient input components framework. This is a high quality patient input because of *x* and *y*. Or this is a low quality and wanting because of *x* and *y*, and try to have a framework for judging that. My experience is that we don't, really (TEC09).

Some technical members noted that patient input would be more helpful and perhaps legitimate if it was more rigorous and critical, reflecting a desire for what Sandman *et al*. (2017: 19) call ‘patient-based evidence’ or ‘gathering data on patients’ perspectives using systematic research’. Some interviewees expressed a desire for patient input that met certain social scientific standards for qualitative evidence, and for patient input to present both the pros and cons of the drug being considered. A number of interviewees mentioned the importance of both lay members and patient input being able to consider tradeoffs: funding one potentially less effective drug would result in less funding available for other therapies (TEC06, TEC21 and TEC26).

In terms of the rigor of patient submissions, one technical member commented on the small number of respondents included in some patient input surveys, saying ‘It's just like a quick back of the envelope survey…sometimes *N* of 8, *N* of 3, once in a while, *N* of 30, but it was on the internet. So the quality of that input…it's tough, right?’. However, the interviewee then gave an example of a particularly compelling piece of information about quality of life that was not available from clinical studies and commented that in some instances, ‘even having that single case is pretty powerful evidence’ (TEC07).

Others expressed concerns about the way patient input is gathered and presented.

I just don't know… if thought is given explicitly to that. To the way in which the voices heard are expressed qualitatively. I certainly don't think there's any theory behind how that information is presented, whereas there's a lot of theory behind how the clinical evidence and the economic evaluation stuff is presented (TEC19).

A low quality submission is a small number of people engaged, anecdotes… That don't have a pros and cons approach to it. The good ones will have perhaps more than one patient advocacy group involved… And for me the ones that are even better are ones that would say ‘we recognize that there are costs involved with this and that for this particular disease problem we need to see savings elsewhere in order to meet the resource requirements needed with a new drug or new technology’ (TEC21).

I would like the patient groups to be a bit more critical of the evidence, if they can be. So I'd like them to sometimes say, we want this, but we don't even know if the quality of life's improved… Or we really want this, but we recognize that it might be super cost-ineffective (TEC05).

Not all interviewees shared these concerns. One commented on the use of surveys in patient input and suggested that even if this type of evidence was different than the standards applied to clinical trials, they were ‘very supportive of the patient contributions’, saying

I guess generally surveys aren't as strong an instrument as trials and so on, but I don't see any other way of doing that… I'm assuming that the information that made it to the table was the best that could be provided (TEC26).

Overall, I observed some ideational change and significant contestation regarding issues about the quality of evidence.

#### 4.2.4 Legitimate inputs: objectivity

Related to concerns about the rigor of patient input, some interviewees also raised concerns about bias. These issues were also present in the early days of public and patient involvement covered by the reports, and suggest a scientific version of legitimate inputs that prioritizes objectivity.

The patient submission information often comes from an advocacy group, that are potentially funded by drug companies, so you can see that they're biased. Or, they're not funded enough to give us information that we're looking for (TEC05).

Frequently, the submissions we get have clearly got industry input in them, and those are really the most worrying because it really doesn't help us in any way, knowing what is really important to the patients and what's not (TEC06).

However, other technical members were less concerned about the possibility of bias in patient input.

So I think… it would be hard for industry to really sway the outcome of this work, really, through the patient voices alone. And I would feel badly on some level about being that skeptical about the patient input that we're hearing simply because there's worries that they're trying to push a pharma agenda (TEC19).

Another member noted that the pharmaceutical industry often funds patient groups but that clinical trials are generally funded by industry, and are still accepted as evidence, and that the same standards should be applied to patient submissions.

If the objective is to turn it into a lobbying process, then I think it works against the [goals of] patient submissions. But if it's done in an open and transparent manner the same way a clinical trial would be done it should receive the same level of receptivity (TEC26).

These ideas were not present in the reports.

#### 4.2.5 Legitimate goals: what makes a ‘good’ recommendation?

Even when technical members acknowledged democratic conceptions of legitimate inputs, they had consistent goals for the scientific outputs of the drug assessment process. There was broad acceptance among interviewees of the idea that patient input could improve recommendations because it may allow the committee to determine which clinical outcomes were valuable. This echoes Staley and Doherty's (2016) findings regarding patient input in England's National Institute of Health and Care Excellence. Essentially, ‘unscientific’ patient input can enhance the committees' ability to analyze clinical evidence. This idea was not present in the earlier reports.

The simple rule is, what does the patient hope the drug will do? So the patient hopes the drug will do *a*, *b*, *c*, and *d*, and then now I'm going look at the clinical evidence, what did the drug do? We use it to prioritize patient relevant outcomes (TEC07).

For many conditions, the patient ultimately needs to be the person to advise: what are the outcomes that are important to them? (TEC09).

If we don't understand what is important to patients, how can we make decisions that make sense? (TEC18).

There was also an acknowledgment that patient input sometimes helped fill gaps in the technical evidence.

a common scenario would be a drug where increasingly evidence from a clinical perspective isn't as robust as we'd like it to be… And in those circumstances for sure, having really meaningful patient impact testimonials and a sense of how this is changing patients lives when they're on them or meeting a need where there really are no other options for therapy, it can be very impactful (TEC19).

I think patient input helps putting evidence into context – in a sense it can legitimize or negate the science rather than is incidental to the science (TEC06f).

Some of these ideas seem to be about making patient input more like the conventional clinical and economic evidence, fitting public and patient involvement into scientific norms of legitimacy. This suggests some change in ideas in that many technical members see patient input as having a specific and necessary role in drug assessment. However, it also potentially sets an unattainable bar for patient input. Patient input is, by definition, from patients themselves or the advocacy groups that purport to represent them. Few advocacy groups have the resources and training to conduct rigorous qualitative research on their constituents, and they may be understandably reluctant to discuss downsides of a drug if they think it will reduce the likelihood of a positive recommendation.

Interviewees and respondents in the reports reported similar scientific goals for drug assessment overall. However, when discussing outputs, some interviewees raised the question of who judges legitimacy, which suggests a democratic element of outputs. When asked whether public and patient involvement contributes to the legitimacy of the committee, a technical member answered, that's a question that can only be answered by those who perceive the work of the committee from the outside … And I think it's all about perception, and so if the perception is that it's not achieving it at the moment then change needs to be made (TEC21).

Others acknowledged the challenges of legitimating negative recommendations. One technical member noted that ‘The main challenge is that recommendations don't necessarily follow what the patients want…the challenge is inherent. But it leads to disappointment from those who provide input. We've changed the format of the recommendations to try to address this, say more about how input is used’ (TEC18). Future research with patient groups will address these questions.

## 5. Discussion and conclusions

Canadian drug assessment began incorporating public and patient involvement in 2006 in response to perceived deficits in its democratic legitimacy. Drug assessment was designed and practiced as a technical, scientific process and it was successful in these terms in the eyes of practitioners and users (public drug plans). However, it did not have provisions to involve other groups affected by its recommendations, namely patients, and by 2006 pressure from patient groups contributed to a policy change (MacPhail and Shea, 2017).

This policy change preceded significant ideational change on the part of existing drug assessment practitioners. In reports from the early years of public and patient involvement, technical members of drug advisory committees were reported to think that existing processes were consistent, fair, objective, transparent and rigorous: in a word, legitimate (Ekos, 2005). This research asked whether there any change in technical members' ideas about legitimacy in drug assessment over time. As a group, have technical members maintained a strictly scientific understanding of legitimacy that prioritizes outputs and cautions against the possibility of bias and less rigorous forms of evidence? Or do technical members' ideas now include concepts related to democratic legitimacy, valuing inclusion and considering a broader range of evidence than in the past?

There is evidence of ideational change, particularly related to processes and inputs to drug assessment. One area where there is broad acceptance of a democratic notion of legitimacy is the role of lay members. Lay members were not discussed specifically in the reports, but their role appears to have become clearer and more important over time. Technical members often discussed lay members' roles in terms of a more democratic process by communicating recommendations to the public and ensuring patient voices are heard in deliberations. Some interviewees have ‘seen a shift in terms of how public input is viewed and incorporated’ (TEC18f), and some are more skeptical, saying

My personal feeling is that those on committees and even those running them do not understand the distinction between processes that attempt to capture social value (democratic legitimacy and reflecting a liberal democratic process) and scientific legitimacy (peer-review and a need for rigour) (TEC07f).

Some of the more critical participant feedback on this research reflected questions about the validity of considering different forms of legitimacy: it was about the premise of the research rather than the findings. ‘I think of the theme of “window dressing” – changing a process to make it look better (whatever that means) versus actually improving the quality of decision-making’ (TEC04f). It is therefore important to note that the experience of ideational change is not universal, and that some technical members remain deeply skeptical about the role for public and patient involvement.

The reports note a lack of consensus about what patient input should contribute to the drug assessment process, but interviews demonstrate more agreement on this. Most interviewees saw patient input as a way to understand which outcomes were valuable to patients. There was an ongoing concern about avoiding bias, and a common thread of how patient input could fit into existing scientific processes. Some frustration with quality of patient input suggests this may not be communicated clearly to submitting groups or is not always feasible.

This research has found that technical members' ideas about inputs and what counts as evidence have shifted incrementally. As a group, they are still interested in the same basic type of information, but are willing to consider ways patients might provide it. Arguments for public and patient involvement from technical members are generally not made in moral terms but rather in scientific terms, which is consistent with maintaining primarily scientific goals for drug assessment. The committee considers patient input not because it is ‘fair’ to do so, but because it improves recommendations. The consistent focus on scientific legitimacy is not surprising: years of training and professional experience outweigh new norms introduced in a policy process that is intense but still only a part of most members' professional lives. However, interviews do demonstrate some flexibility in how legitimate evidence is understood by technical members when they acknowledge the potential for patient input to improve outcomes.

This acceptance of new inputs and processes while maintaining preexisting goals leads me to characterize the change in ideas as bricolage. There is not a complete change in paradigms of legitimacy among technical members, and new processes and inputs do not substitute for existing one, but instead supplement them in complex and sometimes incoherent ways – for example, in the idea that patient input should somehow meet scientific standards for rigor or objectivity.

These findings have implications for how we understand processes of gradual or incomplete ideational change. Identifying change in different parts of the paradigm helps demonstrate that meaningful change in ideas has occurred, even if it at times appears incoherent. It shows that for many technical members, these ideational changes are in fact deep – they go beyond a token acceptance of public and patient involvement to consider how these institutional changes contribute to ‘better’ drug assessment. The findings also suggest how researchers and participants might view the limits of gradual paradigm change. Meaningful changes to ideas about process and input do not necessarily imply changes to ideas about the legitimate goals of drug assessment, a finding that may apply to other policy areas where goals are defined by expert knowledge and provides a foundation for their world view beyond the specific policy considered.

On the practical question of increased legitimacy for drug advisory committees, this research suggests that public and patient involvement has impacted the way committees function, demonstrated by the near-consensus on the value of patient input in providing context for clinical evidence, and the value of lay members in contributing these perspectives in the committees' deliberations. Future research will assess how these roles are perceived by other participants, particularly patient groups, and this research is necessary to determine whether drug assessment has gained legitimacy in the eyes of participants other than technical members. To repeat the words of one interviewee cited above, the question of increased legitimacy ‘can only be answered by those who perceive the work of the committee from the outside’ (TEC_21).

Future research will also critically examine questions about the practical implications of public and patient involvement for the nature and number of positive drug listing recommendations. Measurement of the influence or use of public and patient input is a longstanding challenge in the field (Li *et al*., 2015), and asking technical members if they can recall instances where public and patient involvement resulted in a different recommendation than might have been expected with only technical input is a blunt instrument for measuring impact. Even technical members who could not recall a specific instance when public and patient involvement changed a recommendation were reluctant to dismiss its influence (Author), so future research will explore the links between perceptions that public and patient involvement ‘changes’ recommendations, and perceptions of democratic legitimacy for the process as a whole.

The research also has practical implications for drug assessment processes in other jurisdictions, and raises particularly pressing questions in the context of policy responses to a global pandemic. Patient advocates are already raising concerns about the exclusion of public and patient voices from pandemic response planning in England and Ireland, two countries with robust public and patient involvement in health policy (Murphy *et al*., 2020; Richards and Scowcroft, 2020). The Canadian Agency from Drugs and Technologies in Health, which houses national-level drug assessment and public and patient engagement, has created an evidence portal for COVID-19 that is aimed primarily at scientists and decision-makers (CADTH 2020) and the Canadian National Advisory Committee on Immunization, which makes recommendations about the use of vaccines to the Public Health Agency of Canada, does not have a formal process for public and patient involvement (NACI 2020). It seems likely that Canadians, and citizens of other countries, will demand some form of democratic input or deliberation on difficult questions of how the risks and benefits of a new vaccine are negotiated and how access to future vaccines will be determined. This research suggests that changing participants' ideas about legitimacy to incorporate both scientific and democratic concerns is complex, but not impossible. Understanding these challenges is the first step to address them directly and concretely, and likely the best opportunity for ensuring an effective and equitable policy process.
